# Growth differentiation factor 11 regulates high glucose-induced cardiomyocyte pyroptosis and diabetic cardiomyopathy by inhibiting inflammasome activation

**DOI:** 10.1186/s12933-024-02258-3

**Published:** 2024-05-07

**Authors:** Jing Zhang, Guolong Wang, Yuxuan Shi, Xin Liu, Shuang Liu, Wendi Chen, Yunna Ning, Yongzhi Cao, Yueran Zhao, Ming Li

**Affiliations:** 1 State Key Laboratory of Reproductive Medicine and Offspring Health, Center for Reproductive Medicine, Institute of Women, Children and Reproductive Health, Shandong University, 250012 Jinan, Shandong China; 2 National Research Center for Assisted Reproductive Technology and Reproductive Genetics, Shandong University, 250012 Jinan, Shandong China; 3 Key Laboratory of Reproductive Endocrinology (Shandong University), Ministry of Education, 250012 Jinan, Shandong China; 4 Shandong Technology Innovation Center for Reproductive Health, 250012 Jinan, Shandong China; 5 Shandong Provincial Clinical Research Center for Reproductive Health, 250012 Jinan, Shandong China; 6 Shandong Key Laboratory of Reproductive Medicine, Shandong Provincial Hospital Affiliated to Shandong First Medical University, 250012 Jinan, Shandong China; 7 Research Unit of Gametogenesis and Health of ART-Offspring, Chinese Academy of Medical Sciences (No. 2021RU001), 250012 Jinan, Shandong China

**Keywords:** DCM, Pyroptosis, GDF11, ASC, Cardioprotection, PPARα

## Abstract

**Background:**

Diabetic cardiomyopathy (DCM) is a crucial complication of long-term chronic diabetes that can lead to myocardial hypertrophy, myocardial fibrosis, and heart failure. There is increasing evidence that DCM is associated with pyroptosis, a form of inflammation-related programmed cell death. Growth differentiation factor 11 (GDF11) is a member of the transforming growth factor β superfamily, which regulates oxidative stress, inflammation, and cell survival to mitigate myocardial hypertrophy, myocardial infarction, and vascular injury. However, the role of GDF11 in regulating pyroptosis in DCM remains to be elucidated. This research aims to investigate the role of GDF11 in regulating pyroptosis in DCM and the related mechanism.

**Methods and results:**

Mice were injected with streptozotocin (STZ) to induce a diabetes model. H9c2 cardiomyocytes were cultured in high glucose (50 mM) to establish an in vitro model of diabetes. C57BL/6J mice were preinjected with adeno-associated virus 9 (AAV9) intravenously via the tail vein to specifically overexpress myocardial GDF11. GDF11 attenuated pyroptosis in H9c2 cardiomyocytes after high-glucose treatment. In diabetic mice, GDF11 alleviated cardiomyocyte pyroptosis, reduced myocardial fibrosis, and improved cardiac function. Mechanistically, GDF11 inhibited pyroptosis by preventing inflammasome activation. GDF11 achieved this by specifically binding to apoptosis-associated speck-like protein containing a CARD (ASC) and preventing the assembly and activation of the inflammasome. Additionally, the expression of GDF11 during pyroptosis was regulated by peroxisome proliferator-activated receptor α (PPARα).

**Conclusion:**

These findings demonstrate that GDF11 can treat diabetic cardiomyopathy by alleviating pyroptosis and reveal the role of the PPARα-GDF11-ASC pathway in DCM, providing ideas for new strategies for cardioprotection.

**Supplementary Information:**

The online version contains supplementary material available at 10.1186/s12933-024-02258-3.

## Introduction

Diabetic cardiomyopathy (DCM) describes a series of cardiac structural and functional abnormalities caused by diabetes [1] that is independent of diabetic macrovascular complications and can lead to the development of heart failure [2]. DCM is a condition characterized by myocardial fibrosis, hypertrophy of cardiomyocytes, inflammation, and apoptosis [3]. The various molecular processes involved in DCM are not fully understood, leading to a lack of specific treatment recommendations for individual patients [4]. At present, the treatment options for DCM are limited to lifestyle changes and glycemic management.

Pyroptosis is a recently discovered form of programmed cell death characterized by cell membrane rupture, the release of cellular contents, and the activation of a severe inflammatory response [5]. Inflammasome activation is crucial for pyroptosis [6]. After inflammasome activation, gasdermin D (GSDMD) is cleaved to generate N-terminal peptides that attach to the cell membrane, forming pores. As the number of pores increases, the cell is gradually lysed [5, 7]. The inflammasome is composed of pattern recognition receptors (PRRs), apoptosis-associated speck-like protein containing a caspase recruitment domain (ASC), and pro-caspase-1 [8]. As an adaptor protein for various inflammasomes, ASC contains an N-terminal PYD and a C-terminal caspase recruitment domain (CARD) [9]. ASC is recruited to clustered PYDs of oligomerized NLRP3 molecules through homotypic PYD-PYD interactions, resulting in the formation of speck-like ASC filaments. The C-terminal CARD of filamentous ASC serves as a platform for recruiting the effector protein caspase-1 [9]. Studies have confirmed the crucial significance of pyroptosis in the progression of DCM [10]. In individuals with diabetes, the expression levels of caspase-1, NLRP3, and GSDMD were observed to be increased in the heart [11]. Mishra PK et al. found that the removal of MMP9 can effectively prevent hyperglycemia-induced pyroptosis in hCSCs [12]. Sumit Kar et al. discovered that exercise training can reduce cardiomyocyte pyroptosis, thereby preventing high-fat diet-induced DCM [13]. Therefore, inhibiting cardiomyocyte pyroptosis could potentially slow the progression of DCM.

Growth differentiation factor 11 (GDF11) is a member of the transforming growth factor β superfamily. It is widely expressed in the body, with particularly high levels in the kidneys, spleen, and heart, and plays an important role in various physiological and pathological states [14, 15]. A reduction in serum GDF11 levels is associated with age-related cardiac hypertrophy [16]. A recent study showed that GDF11 can prevent endothelial damage and inhibit the formation of atherosclerotic lesions in apolipoprotein E-null mice [17]. Numerous studies have consistently demonstrated that GDF11 plays a significant role in pyroptosis and the treatment of cardiovascular disease [18–20]. However, the potential role of GDF11 in regulating cardiomyocyte pyroptosis to prevent the progression of diabetic cardiomyopathy has not been experimentally confirmed. In this study, we constructed a mouse model of type 1 diabetes by intraperitoneal injection of streptozotocin (STZ). After injecting STZ, we were able to successfully establish a DCM model four months later. To investigate the effect of altering GDF11 expression on the progression of diabetic cardiomyopathy (DCM), we used adeno-associated virus (AAV)9 to overexpress GDF11.

## Materials and methods

### Animals and experimental protocols

Male C57/BL6J mice (6–8 weeks old, 20–25 g) were obtained from Weitong Lihua Experimental Animal Technology (Beijing, China) and carefully reared in the SPF laboratory of the animal experimental center of Shandong University. The animal experiments were approved by Shandong University’s Ethics Committee on Animal Research (No. 21,172). All animal studies adhere to the guidelines set forth in Directive 2010/63/EU of the European Parliament concerning the protection of animals used for scientific purposes. All mice were housed under pathogen-free conditions at 20–25 °C and 45% humidity on a 12 h light/dark cycle and fed sterile food and drinking water. The mice were divided into the control and DCM groups with a random number table.

Male C57/BL6J mice were fed a high-fat diet (HFD) for 1 month and then intraperitoneally injected with a low dose (60 mg/kg) of STZ (Sigma, St. Louis, MO, United States) dissolved in citrate buffer (pH 4.5) for three consecutive days. After five days, mice with fasting blood glucose (FBG) levels > 16.7mmol/L (measured via a glucose meter from the tail vein) were considered diabetic. After successful establishment of diabetes, diabetic mice received AAV9-GDF11 or AAV9 negative control by tail vein injection every month (each mouse received a total of 60–80 µl AAV9-GDF11 or AAV9-NC [Genechem, Shanghai, China, 5.0–6.5 × 10^13^ viral genomes/mL]). In addition, fenofibrate (MedChemExpress, US, 100 mg/kg), a selective PPARα agonist, was administered to some mice by intraperitoneal injection every day.

The mice were divided into six groups: (1) the negative control (NC) group, (2) the DCM group, (3) the DCM and empty AAV treatment (DCM + AAV9-NC) group, (4) the DCM and AAV9-GDF11 treatment (DCM + AAV9-GDF11) group, (5) the DCM and AAV9-GDF11 and PPARα agonist treatment (DCM + AAV9-GDF11 + PPARα agonist) group, and (6) the DCM and AAV9-NC and PPARα agonist treatment (DCM + AAV9-NC + PPARα agonist) group. After study, all animals were anesthetized with an intraperitoneal injection of sodium pentobarbital (50 mg/kg body weight) and subsequently euthanized by cervical dislocation.

### Echocardiography

Echocardiography was performed for the in vivo assessment of cardiac structure and function. Mice were placed in a 37 °C incubator, and anesthesia was induced with 1.5% inhaled isoflurane and maintained with 0.5% inhaled isoflurane. Vevo 2100 (Visual Sonics, Canada) with a center frequency of 30 MHz Scan was used to detect cardiac motion in the long-axis view as previously described [21]. Then, the probe was rotated 90 degrees to detect cardiac motion in the short-axis view, and graphs were acquired in M-Mode near the papillary muscles. The parameters of left ventricular function measured included the left ventricle internal dimension in systole (LVIDs) and diastole (LVIDd), left ventricular ejection fraction (LVEF), and left ventricular fractional shortening (LVFS), which were calculated by computerized algorithms.

### Cell culture

The H9c2 cells were purchased from American Type Culture Collection. The cell lines were maintained in Dulbecco’s modified Eagle’s medium (DMEM, Invitrogen, 12,100,046) supplemented with 10% fetal bovine serum (FBS, Gbico, United States), 100 U/ml penicillin and 100 µg/ml streptomycin (Beyotime Biotechnology, Shanghai, China) at 37 °C in a 5% CO_2_ humidified atmosphere. Cells were treated with 50mM glucose at the indicated times.

### PI staining

The detailed steps of PI staining were as follows: After treating cardiomyocytes with HG, they were placed on a glass slide and washed with PBS three times. PI working solution was prepared at a concentration of 1.5 μm. The cells were then incubated on ice for 5 min. After another round of washing with PBS, the cells were fixed with 4% PFA on ice for 30 min. Following a quick wash, the cardiomyocytes were mounted with medium containing DAPI and examined using a Nikon Eclipse Ti-S fluorescence microscope. To calculate the percentage of pyroptotic cells, the total number of PI-positive nuclei was divided by the total number of nuclei stained with DAPI.

### RNA interference (RNAi)

The GDF11 RNAi sense sequence was as follows:

si-GDF11-1:

Forward primer (5’ to 3’): CCACAAAGCAACUGGGGAATT

Reverse primer (5’ to 3’): UUCCCCAGUUGCUUUGUGGTT

si-GDF11-2:

Forward primer (5’ to 3’): CAGUGGACUUCGAGGCUUUTT

Reverse primer (5’ to 3’): AAAGCCUCGAAGUCCACUGTT

The ASC RNAi sense sequence was as follows:

si-ASC-1:

Forward primer (5’ to 3’): GCUACUAUCUGGAGGGGUATT

Reverse primer (5’ to 3’): UACCCCUCCAGAUAGUAGCTT

si-ASC-2:

Forward primer (5’ to 3’): GGGCACAGCCAGAACAGAATT

Reverse primer (5’ to 3’): UUCUGUUCUGGCUGUGCCCTT

The PPARα RNAi sense sequence was as follows:

si-PPARα-1:

Forward primer (5’ to 3’): GAACAUCGAGUGUCGAAUATT

Reverse primer (5’ to 3’): UAUUCGACACUCGAUGUUCTT

si-PPARα-2:

Forward primer (5’ to 3’): GGCGAACUAUUCGGCUAAATT

Reverse primer (5’ to 3’): UUUAGCCGAAUAGUUCGCCTT

### Cell transfection with plasmids or siRNAs

A c-Myc-GDF11-overexpressing pcDNA3.1 plasmid, HA-ASC-overexpressing pcDNA3.1 plasmid and PPARα-overexpressing pcDNA3.1 plasmid were purchased from The Beijing Genomics Institute. H9c2 cardiomyocytes were transfected with Lipofectamine 3000 (Invitrogen, Carlsbad, CA, USA) using Opti-MEM reduced serum medium (Gibco, Carlsbad, CA, USA).

### Measurement of serum cytokine levels

Serum was obtained by centrifugation (4 °C, 1,500 *g*, 20 min) of blood collected from the eye sockets of the mice and stored at − 80 °C. After various treatments, the concentrations of IL-1β (EK201B/3-AW1, MultiSciences, Hangzhou, China) and IL-18 (EK218-03, MultiSciences, Hangzhou, China) in the serum of DCM model mice were measured by ELISA kits.

### Histological analysis

After testing the heart function of the mice, the mice were deeply anesthetized using diethyl ether (10%, Buxco Electronics, Inc., Wilmington, NC, USA) and then euthanized to extract blood and collect heart tissue. A portion of each mouse heart was preserved in 4% paraformaldehyde (Biosharp, Hefei, China) for future histopathological analysis, while another portion was frozen in liquid nitrogen for subsequent protein extraction. Myocardial tissues were embedded in paraffin and sectioned to 5 μm. Then, the sections were stained with hematoxylin and eosin (H&E) or Masson’s trichrome to observe changes in myocardial morphology and assess the degree of collagen deposition, respectively.

### H&E staining

H&E staining was carried out following standard protocols. In brief, mouse heart sections were deparaffinized, rehydrated, and then stained with hematoxylin (Beyotime in Beijing, China). After differentiation using acidic ethanol, the sections were stained with eosin (Beyotime in Beijing, China). Finally, the sections were dehydrated and mounted using Permount (Fisher Scientific in Shanghai, China).

### Masson staining

To assess cardiac fibrosis, we conducted Masson trichrome staining using a staining kit (Solarbio, G1340) according to the manufacturer’s instructions. Cardiac fibrosis was visualized as blue Masson’s trichrome staining. The fibrotic area ratio was quantified using ImageJ software by an observer who was unaware of the sample identities.

### Immunohistochemistry

Heart samples were collected and preserved in a solution containing 4% paraformaldehyde. Afterward, the samples were dehydrated and cut into 5 μm thin sections. The sections were then treated with hydrogen peroxide, and antigen repair was performed with citrate buffer (Beyotime, Shanghai, China). The sections were permeabilized with Triton X-100 (Beyotime, Shanghai, China) and blocked using BSA (Sigma‒Aldrich, St. Louis, MO), and the sections were then incubated with primary antibody overnight at 4 °C. Afterward, they were incubated with a secondary antibody labeled with HRP for 30 min at 37 °C. Visualization was performed using a microscope and a DAB Horseradish Peroxidase Color Development Kit. Finally, the sections were sealed with a neutral balsam fixative. The number of positive cells was counted using ImageJ software, and the mean density was quantified using Image-Pro Plus software.

### Immunofluorescence

Cells were fixed and permeabilized with a solution containing 4% paraformaldehyde and 0.1% Triton X-100 at 4 °C for 10 min. To prevent nonspecific binding of antibodies, the cells were incubated with fetal calf serum (5% v/v) for 30 min. Next, the cells were treated with an anti-GDF11 antibody for 2 h at room temperature. After washing the cells three times, they were treated with Alexa Fluor 594-conjugated anti-rabbit secondary antibody for 2 h at room temperature. Then, the cells were treated with an anti-ASC antibody for 2 h at room temperature. Following another round of washing, the cells were treated with Alexa Fluor 488-conjugated anti-rabbit secondary antibody for 2 h at room temperature. Finally, the nuclei of the cells were stained with DAPI. Images of the cells were captured using a Nikon Eclipse Ti-S fluorescence microscope.

Heart samples were collected and preserved in a solution containing 4% paraformaldehyde overnight. They were then dehydrated, sliced into thin sections (5 μm), and treated with hydrogen peroxide followed by heating at 95 °C for 10 min in citrate buffer. The samples were permeabilized with Triton-100 and then blocked using 5% BSA. After incubating the samples overnight with primary antibody at 4 °C, a secondary antibody was applied at 37 °C for 90 min. All immunofluorescence images were taken using a fluorescence microscope. The details of the antibodies used can be found in Supplementary Table 1.

### Western blotting

Protein samples were separated by SDS‒PAGE, transferred to PVDF membranes (Millipore, Beijing, China), which were incubated with different primary antibodies, including anti-GDF11, anti-Gasdermin D, anti-NLRP3, anti-ASC, anti-c-Caspase-1, anti-PPARα, anti-IL-1β and anti-β-Actin antibodies (Supplementary Table 1). The membranes were incubated for 60 min with secondary antibody, and Protein bands were scanned using a ChemiDoc™ XRS + system. β-Actin was used as an internal reference.

### Coimmunoprecipitation assays

Cells were lysed on ice for 30 min in 500 µl of NP-40 lysis buffer, which contained 50 mM Tris-HCl, 150 mM NaCl, 1 mM EDTA, 1% NP-40, 10% glycerol, 0.2 mM PMSF, and protease inhibitor. After lysing the cells, the lysates were cleared of debris by centrifugation. A small portion of the cleared lysates (50 µl) was set aside as the input. The remaining lysates were subjected to immunoprecipitation to isolate specific proteins of interest. Specifically, 2 µg of antibody and 50 µl of Protein-A/G PLUS-Agarose beads were added to the lysates. The samples were then incubated overnight at 4 °C with rotation. Next, the agarose beads were washed three times with low-salt NP40 lysis buffer and twice with high-salt lysis buffer to remove unbound proteins. Finally, the proteins that were bound to the agarose beads were released by boiling the beads in sample buffer. The released proteins were then separated using SDS‒PAGE gels and analyzed using immunoblotting.

### Proteomic analysis

Proteomic analysis (DCM + AAV9-NC and DCM + AAV9-GDF11 groups were performed using mass spectrometry in data-dependent acquisition mode. To designate significant changes in protein expression, a fold-change of > 1.2 or < 0.83 and a P-value of < 0.05 using Student’s t-test were set as cut-off values. The differential proteins were mapped to the KEGG database (https://www.kegg.jp/kegg/pathway.html) to enrich KEGG pathways.

### Data analysis and statistics

The results are expressed as the mean ± SEM of a minimum of three separate experiments. All significance tests were performed by Student’s t-test. The variation among the different groups was assessed using a statistical analysis called one-way ANOVA followed by Tukey’s post hoc test. A significance level of *p* < 0.05 was used to determine if the differences observed were statistically significant.

## Results

### GDF11 was down-regulated in DCM mice heart and cardiomyocytes

**Fig. 1:**
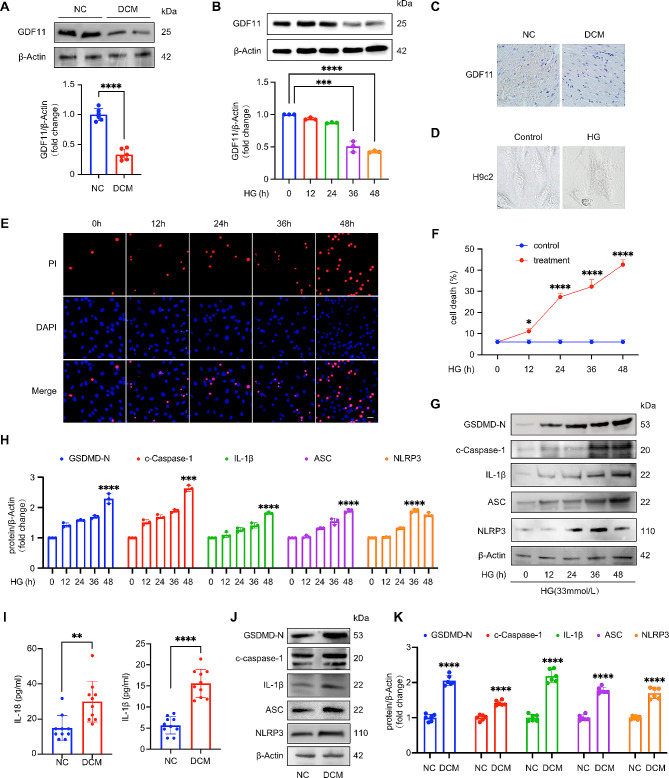
Downregulation of GDF11 expression and pyroptosis in diabetic mouse heart and cardiomyocytes treated with high glucose. (**A**) Western blot analysis of GDF11 protein level in mice hearts from NC and DCM models (*n* = 6 per group). (**B**) GDF11 protein levels in cardiomyocytes treated with high glucose for the indicated time were detected by Western blotting (*n* = 3 per group). (**C**) Immunohistochemistry of GDF11 on DCM mice heart. Scale bar, 50 μm (*n* = 5 per group). (**D**) Morphological alterations in cells cultured with high glucose were indicative of pyroptosis, characterized by swelling and rupture of the cell membrane. Scale bar, 10 μm. (**E**–**F**) PI assay of H9c2s treated with high glucose for 0 to 48 h. Scale bar, 50 μm (*n* = 3 per group). (**G**–**H**) Western blot analysis of pyroptosis-associated proteins (GSDMD-N, c-Caspase-1, IL-1β, ASC, NLRP3) from H9c2s treated with high glucose for 0 to 48 h (*n* = 3 per group). (**I**) ELISA results showing the levels of interleukin-18 (IL-18) and interleukin-1 beta (IL-1β) present in the mice serum from NC and DCM models (*n* = 10 mice per group). (**J**) Representative western blot bands for pyroptosis-associated proteins (GSDMD-N, c-Caspase-1, IL-1β, ASC, NLRP3). (**K**) Western blot analysis of GSDMD-N, c-Caspase-1, IL-1β, ASC, NLRP3 in mice hearts from NC and DCM models (*n* = 6 per group). Data are presented as means ± standard deviation from at least three independent experiments. *P < 0.05, **P < 0.01, ***P < 0.001, ****P < 0.0001. Statistical analysis was carried out by Student’s t-test

First, we explored the expression of GDF11 in the hearts of DCM mice. As shown in Fig. 1A, the protein level and activity of GDF11 were decreased in the hearts of mice with DCM. Immunostaining also revealed that the expression of GDF11 was decreased in cardiomyocytes in the hearts of mice with DCM (Fig. 1C). In order to create a more realistic simulation of diabetic cardiomyopathy, we treated H9c2 cells with DMEM culture medium containing 50mM glucose. We observed cellular membrane swelling and rupture in the H9c2 cells (Fig. 1D), indicating the involvement of pyroptosis in the process of DCM. Furthermore, we observed that with increasing duration of high-glucose treatment, the expression of GDF11 in H9c2 cardiomyocytes gradually decreased (Fig. 1B), which is consistent with the results observed in the in vivo model. These findings suggest that GDF11 may help protect against the development of DCM.

### Pyroptosis in diabetic mouse cardiomyocytes and high glucose-treated H9c2 cells

Based on the morphological changes of H9c2 cells after high glucose treatment, we further investigated whether cardiomyocytes in a long-term diabetic state truly undergo pyroptosis. In vitro model, we treated H9c2 cardiomyocytes with 50mM glucose for 0–48 h and observed an increase in the number of PI-positive cells as the duration of high-glucose treatment increased (Fig. 1D and E).

WB results displayed that the expression of pyroptosis-related proteins (GSDMD-N, c-Caspase-1, IL-1β, ASC, and NLRP3) increased (Fig. 1G and H). We observed that compared to normal mice, diabetic mice showed increased serum levels of the pyroptosis-related inflammatory factors IL-1β and IL-18 (Fig. 1I). Meanwhile, we found increased expression of pyroptosis-related proteins (GSDMD-N, c-Caspase-1, IL-1β, ASC, and NLRP3) in the hearts of diabetic mice compared to normal mice (Fig. 1J and K). These findings indicate that DCM is associated with cardiomyocyte pyroptosis.

### High glucose-induced pyroptosis in cardiomyocytes is dependent on inflammasome activation

Inflammasome activation is the most common mechanism underlying pyroptosis and largely relies on the bridging function of the adaptor protein ASC [22]. When external stimuli are present, ASC translocates and attaches to a recognition receptor, facilitating the assembly of the inflammasome.

**Fig. 2:**
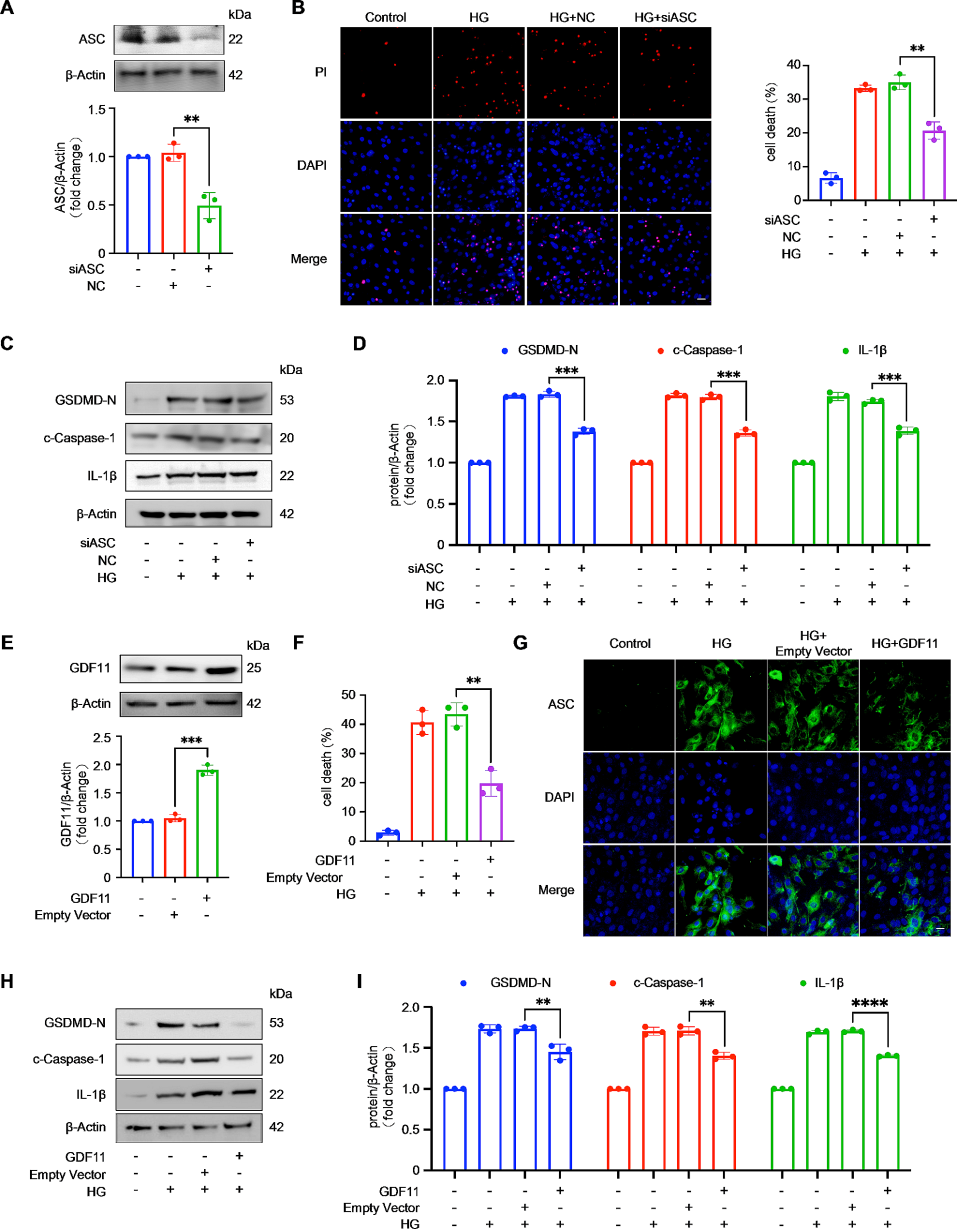
GDF11 inhibits pyroptosis, that is reliant on the activation of the inflammasome, in high-glucose-treated H9c2 cells. (**A**) ASC was knocked down by using the small interfering RNA (*n* = 3 per group). Knockdown of ASC prevented pyroptosis in cardiomyocytes exposed to high glucose for 36 h compared with the control. Pyroptosis was detected by PI assay (**B**) (*n* = 3 per group) and western blot analysis of pyroptosis-associated proteins (GSDMD-N, c-Caspase-1, IL-1β, ASC, NLRP3) (**C**–**D**) (*n* = 3 per group). Scale bar, 50 μm; (**E**) Forced expression of GDF11 using the eukaryon expression plasmid with EF1α promoter in cardiomyocytes (*n* = 3 per group). Forced expression of GDF11 prevented pyroptosis in cardiomyocytes exposed to high glucose for 36 h compared with the control. Pyroptosis was detected by PI assay (**F**) (*n* = 3 per group) and western blot analysis of pyroptosis-associated proteins (GSDMD-N, c-Caspase-1, IL-1β, ASC, NLRP3) (**H**–**I**) (*n* = 3 per group); (**G**) Immunofluorescence staining was used to detect the expression of ASC in H9c2 cardiomyocytes (*n* = 3 per group). Scale bar, 50 μm. Data are presented as means ± standard deviation from at least three independent experiments. **P < 0.01, ***P < 0.001, ****P < 0.0001. Statistical analysis was carried out by Student’s t-test

To investigate whether high blood glucose levels induce cardiomyocyte pyroptosis through inflammasome activation, we first examined expression changes in ASC and NLRP3. The results showed that ASC and NLRP3 expression increased in both the in vivo and in vitro models (Fig. 1G and J). Next, we induced ASC knockout (Fig. 2A) and found that the number of PI-positive cells decreased in ASC knockout cells after HG stimulation (Fig. 2B). ASC silencing also reduced the expression of pyroptosis-related proteins (GSDMD-N, c-Caspase-1, and IL-1β) (Fig. 2C and D). To enhance the persuasiveness of our findings, we further overexpressed ASC in H9c2 cells using an overexpression plasmid (Figure S1A). The results showed that when ASC was overexpressed there was an increase in the number of PI-positive cells after HG stimulation (Figure S1B), and overexpression of ASC also increased the expression of pyroptosis-related proteins (Figure S1C and S1D).

### GDF11 inhibits pyroptosis in high glucose-treated H9c2 cells 

Then, we began to investigate the role of GDF11 in cardiomyocytes. We overexpressed GDF11 in H9c2 cells by transfection with a GDF11 expression plasmid (Fig. 2E). Overexpression of GDF11 reduced the proportion of PI-positive cells after HG stimulation (Fig. 2F) and decreased the expression of pyroptosis-related proteins (Fig. 2H and I). This suggests a negative correlation between GDF11 expression and pyroptosis. Interestingly, we found that overexpression of GDF11 inhibited the increase in ASC expression induced by HG treatment (Fig. 2G), suggesting that GDF11 inhibits pyroptosis by suppressing inflammasome activation. Next, GDF11 was knocked down in H9c2 cells by transfection with GDF11 small interfering RNA (Figure S1E). An increase in the number of PI-positive cells was observed in GDF11 knockdown cells after HG stimulation (Figure S1F), and GDF11 silencing also increased the expression of pyroptosis-related proteins (Figure S1G and S1H).

### GDF11 inhibits high glucose-induced pyroptosis of H9c2 cells by binding to ASC

**Fig. 3:**
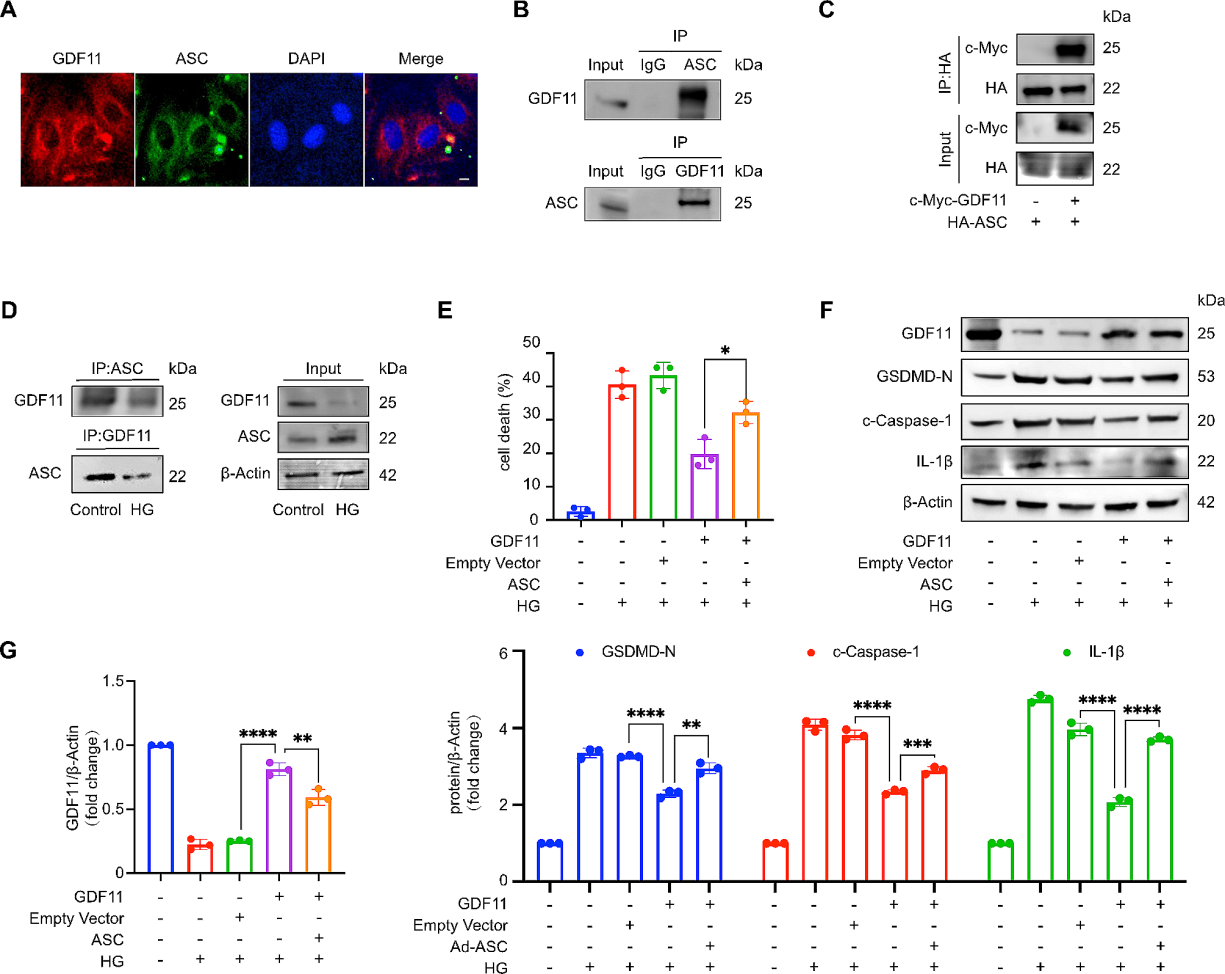
GDF11 inhibits high glucose-induced pyroptosis of H9c2 cells by binding to ASC. (**A**) Colocalization of GDF11 with ASC in cardiomyocytes was detected by immunofluorescence staining. GDF11, red; ASC, green; nuclei, blue; scale bar, 20 μm. (*n* = 3 per group). (**B**) Interaction of endogenous GDF11 with ASC in cardiomyocytes was detected by immunoprecipitation. (**C**) Interaction of GDF11 with ASC was detected by immunoprecipitation in 293T cells with ectopic expression of c-Myc-GDF11 and HA-ASC (*n* = 3 per group). (**D**) Immunoprecipitation assay for GDF11 and ASC in the control and HG group (*n* = 3 per group). (**E**–**G**) Overexpression of GDF11 inhibited pyroptosis in cardiomyocytes, which was abolished by simultaneous overexpression of ASC in cardiomyocytes exposed to high glucose for 36 h. Pyroptosis was detected by PI assay (**E**) (*n* = 3 per group) and western blot analysis of GDF11 and pyroptosis-associated proteins (GSDMD-N, c-Caspase-1, IL-1β, ASC) (**F**–**G**) (*n* = 3 per group). Data are presented as means ± standard deviation from at least three independent experiments. *P < 0.05, **P < 0.01, ***P < 0.001, ****P < 0.0001. Statistical analysis was carried out by Student’s t-test

Next, to gain a deeper understanding of the relationship between GDF11 and ASC, we performed colocalization analysis of GDF11 (red) and ASC (green) in H9c2 cardiomyocytes and observed that both ASC and GDF11 were located in the cytoplasm (Fig. 3A). This led us to suspect that there may be some kind of binding relationship between the two. To confirm this hypothesis, we conducted immunoprecipitation experiments on GDF11 and ASC, both endogenously (Fig. 3B) and exogenously (Fig. 3C). The results showed that GDF11 and ASC can indeed interact with each other. We speculate that under resting conditions or prior to external stimulation, GDF11 and ASC are in a bound state, but when external stimuli are present, their binding decreases, and ASC is released to participate in inflammasome activation. Therefore, we studied the changes in the binding of GDF11 and ASC under high-glucose treatment, and the results were consistent with our hypothesis, as HG treatment decreased the binding of GDF11 to ASC (Fig. 3D).

To confirm that ASC is a target of GDF11, rescue experiments in which ASC and GDF11 were both overexpressed were conducted. Overexpression of GDF11 reduced the proportion of PI-positive cells after HG stimulation (Fig. 3E) and decreased the expression of pyroptosis-related proteins (Fig. 3F and G). However, overexpression of ASC inhibited these trends. In conclusion, ASC may serve as a target of GDF11 in the progression of DCM, GDF11 inhibits high glucose-induced H9c2 cells pyroptosis by binding to ASC.

### GDF11 ameliorates cardiac dysfunction, fibrosis, and pyroptosis in DCM model mice

**Fig. 4:**
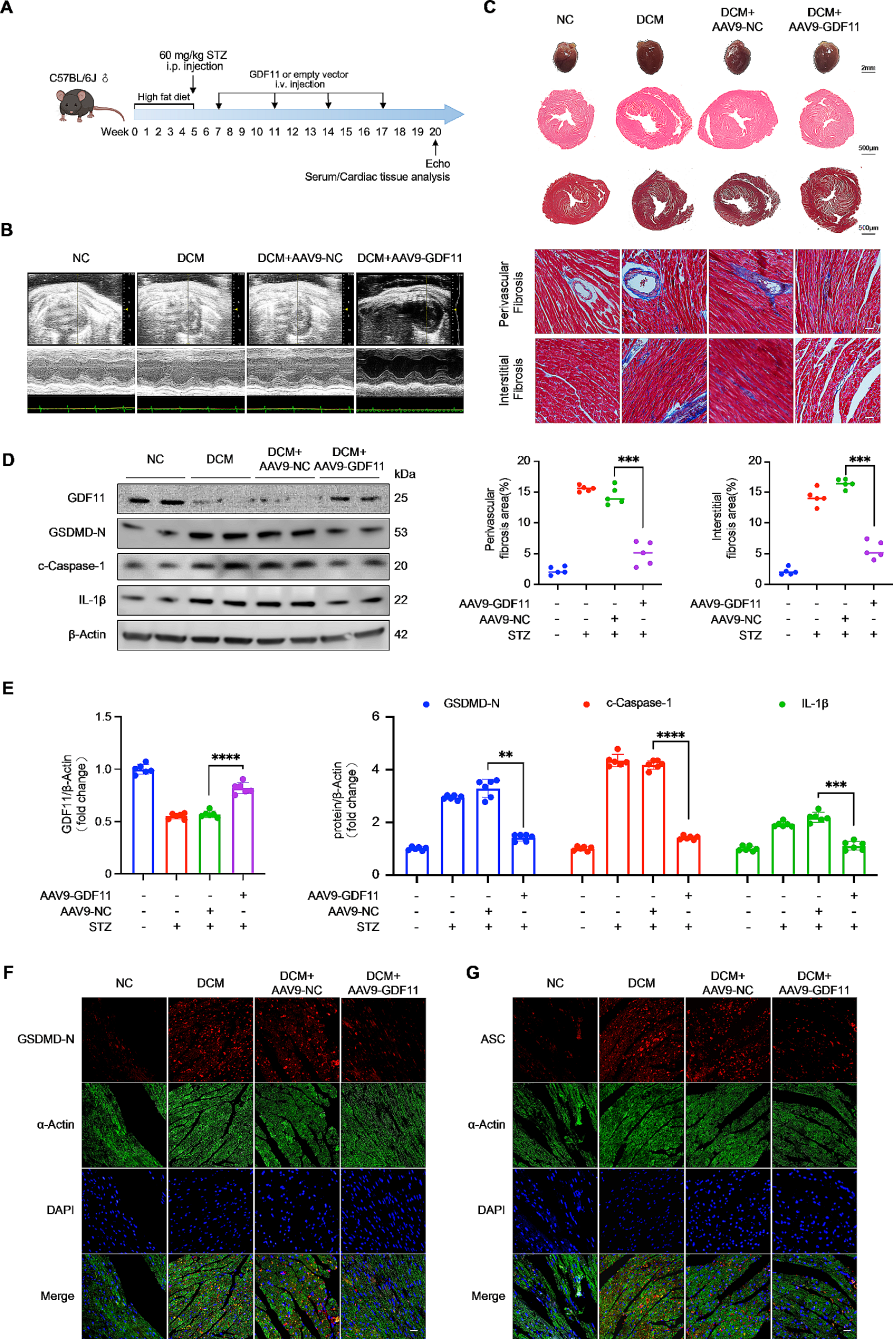
GDF11 ameliorates cardiac dysfunction, fibrosis, and pyroptosis in DCM mice. (**A**) A schematic image showing the study design, doses, and injection schedule. (**B**) Representative images of M-mode echocardiography. (**C**) Representative photographs of the hearts from four groups of mice (the first row, scale bar, 2 mm), cross-sectional images of hematoxylin and eosin (H&E) staining at the papillary muscle level of the hearts (the second row, scale bar, 500 μm), cross-sectional images of Masson staining at the papillary muscle level of the hearts (the third row, scale bar, 500 μm) and Masson-stained sections of hearts from four groups of mice (the Fourth and fifth rows, scale bar, 50 μm) (*n* = 5 per group). (**D**–**G**) The GDF11 overexpression inhibited the pyroptosis of heart tissue. Pyroptosis was detected by western blot analysis (**D**–**E**) (*n* = 6 per group) and immunofluorescence (**F**–**G**) (*n* = 5 per group) of pyroptosis-associated proteins. Pyroptosis-associated proteins, red; α-Actin, green; nuclei, blue; scale bar, 20 μm. Data are presented as means ± standard deviation from at least three independent experiments. **P < 0.01, ***P < 0.001, ****P < 0.0001. Statistical analysis was carried out by Student’s t-test. Data are presented as means ± standard deviation from three independent experiments

To evaluate the role of the GDF11 protein in DCM, we successfully induced diabetes in mice using STZ injection for three consecutive days. After a week, we overexpressed GDF11 by weekly intravenous injection of AAV9-GDF11 to generate mice GDF11 overexpression and diabetic DCM (Fig. 4A). Following AAV9 injection, the heart showed high levels of GDF11 protein (Fig. 4D). While DCM mice exhibited elevated blood glucose levels, overexpression of GDF11 did not affect blood glucose levels in the context of DCM (Supplementary Table 2).

One week prior to sacrifice, the cardiac function of the 4 groups of mice was analyzed. We observed that the DCM model mice had poorer heart function than the NC group mice (Fig. 4B). This was evidenced by a decrease in LVEF, FS, LV mass, and heart rate, as well as an increase in LVIDd, IVSd and LVPW (Table 1). The echocardiography results showed that compared to DCM + AAV9-NC group mice, the mice in the DCM + AAV9-GDF11 group exhibited alleviated heart dysfunction (Fig. 4B; Table 1). According to the results of HE and Masson staining, the hearts of DCM + AAV9-GDF11 group mice were smaller and had reduced myocardial fibrosis compared to those of DCM + AAV9-NC group mice (Fig. 4C). Immunohistochemical staining for collagenase III also demonstrated that GDF11 reduced myocardial fibrosis caused by DCM (Figure S2A). These findings suggest that GDF11 can effectively alleviate DCM-induced cardiac hypertrophy and myocardial fibrosis. Next, we extracted protein from mouse heart tissue, and the Western blotting results showed decreased expression of pyroptosis-related proteins in the hearts of DCM + AAV9-GDF11 group mice compared to those of DCM + AAV9-NC group mice (Fig. 4D and E). Immunofluorescence and immunohistochemical staining of pyroptosis-related proteins also demonstrated that GDF11 inhibited pyroptosis in the hearts of DCM model mice (Fig. 4F–G and Figure S2B-S2F).

**Table 1:** Echocardiographic assessment of left ventricle structural and functional data in mice

	NC	DCM	DCM+ AAV9-NC	DCM+ AAV9-GDF11	DCM+ AAV9-GDF11+ PPARα agonist	DCM+ AAC9-NC+ PPARα agonist
LVEF (%)	83.17 ± 4.42	59.12 ± 6.87^*^	61.11 ± 7.02	79.84 ± 5.41^#^	70.47 ± 5.97^&%^	47.36 ± 10.91^#^
FS (%)	49.24 ± 4.44	26.93 ± 4.85^*^	31.96 ± 4.11	43.37 ± 3.96^#^	37.65 ± 3.56^&%^	26.71 ± 5.87^#^
LVIDd (mm)	2.56 ± 0.20	3.16 ± 0.19^*^	3.22 ± 0.29	2.68 ± 0.13^#^	3.12 ± 0.26^&%^	3.81 ± 0.32^#^
IVSd (mm)	0.80 ± 0.06	1.24 ± 0.06^*^	1.21 ± 0.07	0.89 ± 0.07^#^	1.05 ± 0.05^&%^	1.33 ± 0.14^#^
LV mass (mg)	92.05 ± 15.88	41.04 ± 8.75^*^	39.45 ± 10.45	68.09 ± 13.52^#^	56.09 ± 4.62^&%^	43.15 ± 14.93^#^
LVPW (mm)	0.87 ± 0.04	1.50 ± 0.18^*^	1.30 ± 0.16	0.97 ± 0.08^#^	1.30 ± 0.27^&%^	1.83 ± 0.11^#^
Heart rate(bpm)	445.14 ± 27.81	368 ± 13.52^*^	365.71 ± 12.72	411.71 ± 7.99^#^	442 ± 18.38^&%^	368.71 ± 20.95^#^

P values were calculated using a one-way analysis of variance test and LSD test was used for multiple comparisons (n= 7–10 mice per group). Data are expressed as the mean ± SD

*P < 0.05 vs. NC;^ #^P < 0.05 vs. DCM+AAV9-NC; ^%^P < 0.05 vs. DCM+AAV9-GDF11; ^&^P < 0.05 vs. DCM+ AAV9-NC+PPARα agonist

### PPARα inactivates GDF11 expression and indirectly regulates pyroptosis in cardiomyocytes

**Fig. 5:**
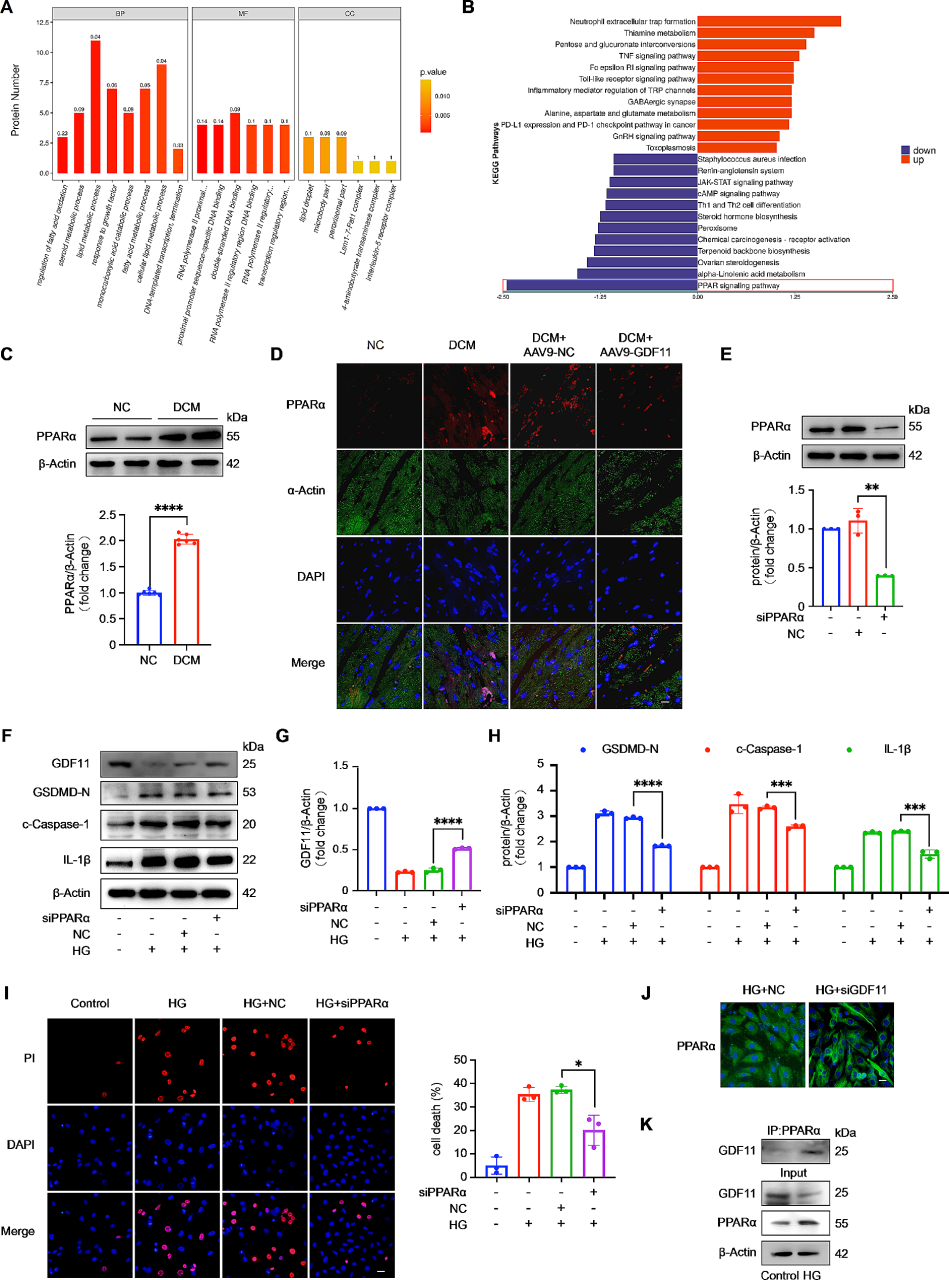
PPARα inactivates GDF11 expression and indirectly regulates pyroptosis of cardiomyocytes. (**A**) GO (Gene Ontology) analysis of differentially expressed proteins based on proteomics analysis. (B) KEGG pathway enrichment analysis of differentially expressed proteins based on proteomics analysis. (**C**) Western blot analysis of PPARα protein level in mice hearts from NC and DCM models (*n* = 6 per group). (**D**) Immunofluorescence staining was used to detect the expression of PPARα in mice heart (*n* = 5 per group). (**E**) PPARα was knocked down by using the small interfering RNA (*n* = 3 per group). (**F**) Representative western blot bands for GDF11 and pyroptosis-associated proteins (NLRP3, ASC, c-Caspase-1, GSDMD-N). Knockdown of PPARα inhibited pyroptosis and increase the decrease of GDF11(**G**) expression in cardiomyocytes exposed to high glucose for 36 h (*n* = 3 per group). Pyroptosis was detected by western blot analysis of pyroptosis-associated proteins (**F** and **H**) and PI assay (**I**) (*n* = 3 per group). (**J**) Immunofluorescence staining was used to detect the expression of PPARα in H9c2 cardiomyocytes (*n* = 3 per group). (**K**) Immunoprecipitation assay for GDF11 and PPARα in the control and HG group (*n* = 3 per group). Data are presented as means ± standard deviation from at least three independent experiments. *P < 0.05, **P < 0.01, ***P < 0.001, ****P < 0.0001. Statistical analysis was carried out by Student’s t-test

To further investigate the mechanism by which GDF11 regulates DCM, we conducted proteomic analysis of the hearts of DCM + AAV9-NC and DCM + AAV9-GDF11 group mice. The results showed that overexpression of GDF11 regulated fatty acid oxidation, fatty acid metabolism, steroid metabolism, and lipid metabolism in DCM model mice (Fig. 5A). KEGG pathway analysis revealed significant inhibition of the peroxisome proliferator-activated receptor (PPAR) signaling pathway (Fig. 5B). Within the PPAR signaling pathway, we focused on PPARα. Many studies have indicated that abnormal elevation of heart PPARα expression is considered an important factor in the development of DCM [23]. Overexpression of PPARα in mice leads to severe cardiomyopathy [24], while inhibition of PPARα prevents the progression of DCM [25, 26]. A significant function of PPARα in the heart is to control the expression of specific genes related to energy metabolism using different mechanisms, such as transactivation or transrepression [23].

We indeed found a decrease in the expression of PPARα in the hearts of DCM model mice (Fig. 5C). Immunofluorescence showed a decrease in PPARα expression in the hearts of DCM + AAV9-GDF11 group mice compared to those of DCM + AAV9-NC group mice (Fig. 5D), indicating a possible regulatory relationship between GDF11 and PPARα. To validate this hypothesis, we investigated the function of PPARα in H9c2 cardiomyocytes. PPARα was knocked down by transfecting H9c2 cells with PPARα siRNA (Fig. 5E). Among PPARα-silenced cells after HG stimulation, a decrease in the number of PI-positive cells was observed (Fig. 5I), and PPARα silencing reduced the expression of pyroptosis-related proteins while increasing GDF11 expression (Fig. 5F and H). This suggests a positive correlation between the expression of PPARα and pyroptosis. Overexpression of PPARα (Figure S3A) was found to decrease the expression of GDF11 during high-glucose treatment and increase the number of PI-positive cells (Figure S3B) and the expression of pyroptosis-related proteins (Figure S3C and S3D), providing support for our hypothesis. Furthermore, we found that the expression of PPARα increased in GDF11 knock down cells after HG stimulation, further supporting our hypothesis (Fig. 5J). The mutual inhibitory relationship between GDF11 and PPARα easily leads to speculation about whether there is a direct or indirect interaction between the two. Therefore, we used immunoprecipitation experiments to detect the binding of GDF11 and PPARα in the control group and the HG group (Fig. 5K). We found that their binding increased under high glucose treatment, indicating that their mutual inhibitory relationship is mediated through binding.

### PPARα reverses the ameliorative effects of GDF11 on cardiac dysfunction, fibrosis and pyroptosis in the hearts of DCM mice

**Fig. 6:**
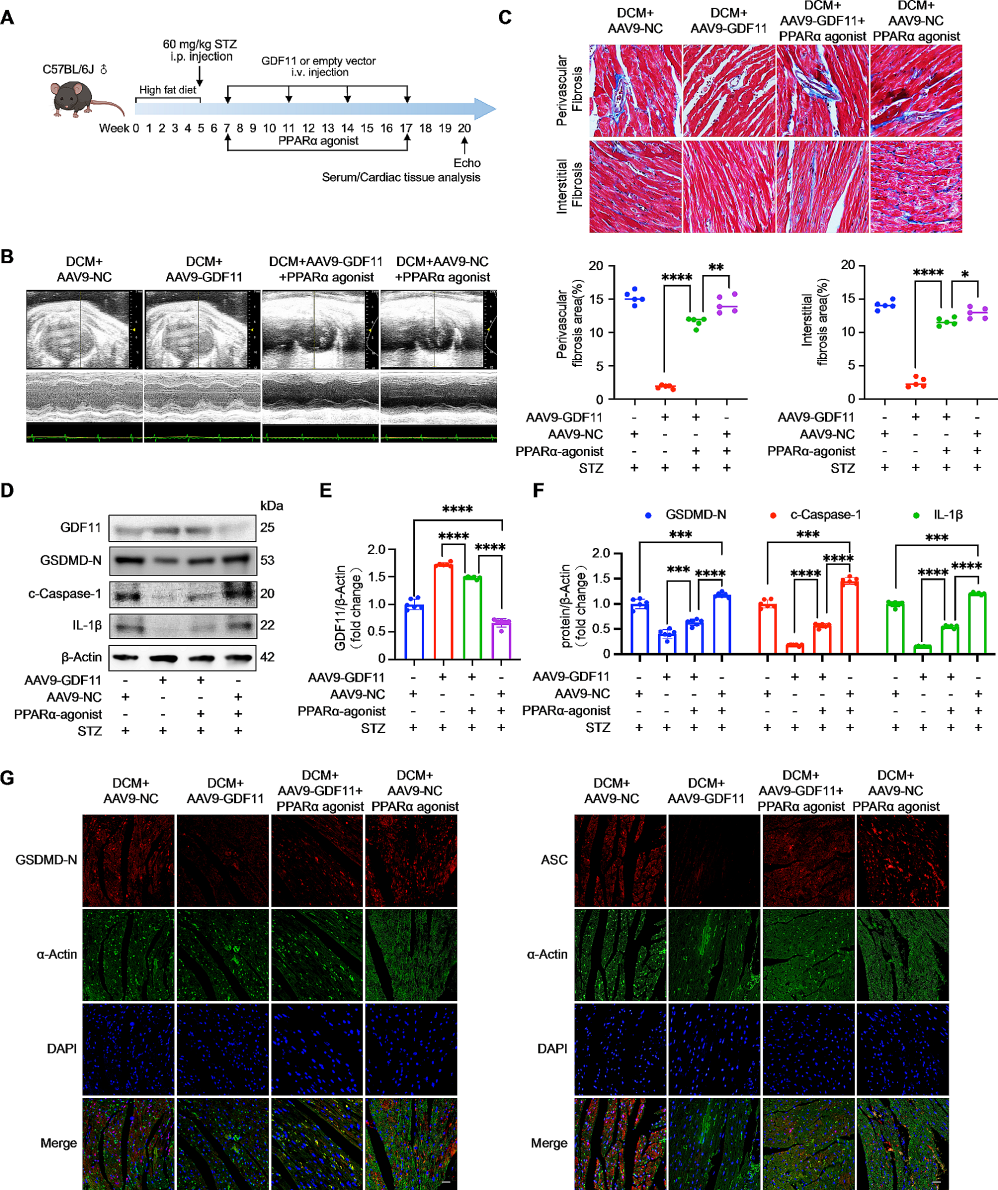
PPARα reverses the ameliorative effects of GDF11 on cardiac dysfunction, fibrosis and pyroptosis in the hearts of DCM mice. (**A**) A schematic image showing the study design, doses, and injection schedule. (**B**) Representative images of M-mode echocardiography. (**C**) Collagen deposition detected by Masson staining (*n* = 5 per group). (**D**) PPARα reverses the ameliorative effects of GDF11 (**E**) on pyroptosis in the hearts of DCM mice (*n* = 6 per group). Pyroptosis was detected by western blot analysis (**F**) (*n* = 6 per group) and immunofluorescence (**G**) (*n* = 5 per group) of pyroptosis-associated proteins. pyroptosis-associated proteins, red; α-Actin, green; nuclei, blue; scale bar, 20 μm. Data are presented as means ± standard deviation from at least three independent experiments. *P < 0.05, **P < 0.01, ***P < 0.001, ****P < 0.0001. Statistical analysis was carried out by Student’s t-test

To further investigate the function and mechanistic connection between PPARα and GDF11 in DCM, we simultaneously administered AAV9-GDF11 and daily intraperitoneal injections of a PPARα agonist to DCM group mice and DCM + AAV9-GDF11 group mice (Fig. 6A). One week before sacrifice, we conducted cardiac function analysis. Echocardiography showed that compared to DCM + AAV9-GDF11 group mice, DCM + AAV9-GDF11 + PPARα agonist group mice had poorer cardiac function (Fig. 6B). This was evident by a decreased LVEF, FS, LV mass, and heart rate, as well as an increased LVIDd, IVSd, and LVPW (Table 1). In comparison to the DCM + AAV9-NC + PPARα agonist group, the DCM + AAV9-GDF11 + PPARα agonist group alleviation of cardiac function impairment (Fig. 6B; Table 1). Furthermore, when compared to the DCM + AAV9-NC group, DCM + AAV9-NC + PPARα agonist group showed exacerbated cardiac function impairment (Fig. 6B; Table 1). The results of Masson’s staining and immunohistochemical staining of collagenase III showed that compared to DCM + AAV9-GDF11 group mice, DCM + AAV9-GDF11 + PPARα agonist group mice had increased myocardial fibrosis (Fig. 6C and Figure S4A). On the other hand, compared to DCM + AAV9-NC + PPARα agonist group mice, DCM + AAV9-GDF11 + PPARα agonist group mice had reduced myocardial fibrosis (Fig. 6C). These findings suggest that PPARα activation exacerbates cardiac functional impairment and myocardial fibrosis in DCM, while overexpression of GDF11 can alleviate the effects of PPARα activation. Next, we extracted protein from mouse heart tissue. The Western blotting results showed that compared to DCM + AAV9-GDF11 group mice, DCM + AAV9-GDF11 + PPARα agonist group mice had increased expression of pyroptosis-associated proteins in the heart (Fig. 6D and E). Compared to DCM + AAV9-NC + PPARα agonist group mice, the expression of pyroptosis-associated proteins in the hearts of DCM + AAV9-GDF11 + PPARα agonist group mice were decreased (Fig. 6D and F). This indicates that PPARα activation exacerbates cardiomyocytes pyroptosis in DCM model mice, and overexpression of GDF11 can alleviate the effects of PPARα activation. Immunofluorescence staining of pyroptosis-associated proteins also showed that GDF11 ameliorate the exacerbation of myocardial pyroptosis caused by PPAR activation in DCM model mice (Fig. 6G and Figure S4B).

## Discussion

DCM involves various physiological alterations, including a reduction in nitric oxide levels, elevated reactive oxidative stress (ROS) levels, the accumulation of collagen, fibrosis, cellular death (apoptosis, necrosis, and autophagy), and inflammation [27, 28]. The study we conducted focused on investigating the different types of infiltrating inflammatory cells and inflammation associated with pyroptotic cell death in a type 1 diabetic model. Additionally, we also investigated the mechanism by which GDF11 is involved in pyroptosis in DCM.

Cardiac dysfunction in DCM is typically caused by abnormal changes in the structure of the myocardium, which is characterized by the accumulation of collagen in the extracellular space and remodeling of the matrix [29]. We observed significant collagen deposition and myocardial fibrosis in the hearts of STZ-induced type 1 diabetes model mice. This pathological change is accompanied by a decrease in GDF11 levels. Previous studies have reported that GDF11 is involved in protecting against various cardiovascular diseases [19, 30–32] and can alleviate diabetic cardiomyopathy by inhibiting apoptosis and oxidative stress [33]. GDF11 can also directly and effectively block the development of diabetes [34, 35]. However, the role of GDF11 in cardiomyocyte pyroptosis in DCM is still unclear. Previous research has already established a connection between GDF11 and pyroptosis in other diseases. It has been reported that GDF11 can ameliorate experimental colitis by inhibiting NLRP3 inflammasome activation [18]. Another study demonstrated that GDF11 plays an antipyroptotic role via the HOXA3/NLRP3 axis to improve heart function in MI [20]. Therefore, we attempted to investigate the specific mechanism by which GDF11 regulates pyroptosis in DCM. To do this, we overexpressed GDF11 in the hearts of DCM mice by intravenous injection of AAV9-GDF11. In mice with STZ-induced diabetes, we observed impaired heart function and evident myocardial pyroptosis in the heart. However, overexpression of GDF11 in diabetic mice resulted in significant improvements in heart function and alleviation of myocardial pyroptosis. To further support this finding, we simulated the environment of DCM by subjecting H9c2 myocardial cells to high-glucose treatment and verified the role of GDF11 in this process. Similarly, GDF11 was found to inhibit high glucose-induced cardiomyocyte pyroptosis.

Pyroptosis is an inflammation-dependent form of programmed cell death often associated with inflammasome activation [6]. The NLR protein, also known as nod-containing protein-like sensors, acts as a receptor for various signals. Inflammasomes, which are categorized based on the inclusion of different sensors, include the NLRP1, NLRP3, NLRC6, NLRC10, and NLPC12 inflammasomes [36]. Many inflammasomes attract the ASC adaptor molecule, which consists of a CARD and a PYD, through interactions of the same type [37]. ASC molecules interact through CARD-CARD and PYD-PYD interactions until all ASC molecules accumulate in one area. The attraction of procaspase-1 to the ASC focus through the CARD-CARD interaction leads to the formation of dimers and the activation of procaspase-1 through self-cleavage, resulting in the production of p10 and p20 subunits [36]. We observed that pyroptosis in the hearts of DCM model mice occurs through inflammasome activation, and we speculate that multiple inflammasomes, including the NLRP3 inflammasome, are involved in this process. Indeed, overexpression of GDF11 can suppress pyroptosis by inhibiting inflammasome activation both in vivo and in vitro. Our research focused on the adaptor protein ASC, which is necessary for inflammasome activation. We found that knocking down ASC significantly alleviated pyroptosis in cardiomyocytes treated with high glucose. Therefore, we attempted to investigate whether there is an interaction between GDF11 and ASC. In our study, we confirmed that GDF11 could directly bind to ASC in cardiomyocytes. Under HG stimulation, the expression of ASC increased, and the expression of GDF11 decreased, leading to a decrease in the GDF11-ASC interaction.

We attempted to investigate whether there are other mechanisms by which GDF11 regulates DCM. To do so, we collected mouse hearts from the DCM + AAV9-GDF11 and DCM + AAV9-NC groups for proteomic analysis. The results of the proteomic analysis showed that overexpression of GDF11 regulated fatty acid oxidation and metabolism, steroid metabolism, and lipid metabolism in DCM model mice (Fig. 5A). Additionally, overexpression of GDF11 significantly inhibited the PPAR signaling pathway (Fig. 5B). Previous studies have reported that increased expression of PPARα is crucial for the progression of DCM [23]. PPARα can participate in the regulation of DCM through various mechanisms, including mitochondrial biogenesis [38], mitochondrial fatty acid and glucose oxidation [24, 39, 40], and branched chain amino acid (BCAA) metabolism [41]. We aimed to investigate the involvement of PPARα in high glucose-induced cardiomyocyte pyroptosis and DCM by regulating the expression of GDF11. We injected PPARα agonists into DCM + AAV9-GDF11 and DCM + AAV9-NC group mice (Fig. 6A) and found that the activation of PPARα exacerbated DCM and inhibited the protective effect of GDF11 against DCM. In HG-treated H9c2 cardiomyocytes, knocking down PPARα not only increased the expression of GDF11 but also reduced HG-induced pyroptosis. These results suggest that PPARα can participate in the regulation of DCM and HG-induced cardiomyocyte pyroptosis by regulating the expression of GDF11. We have made a novel discovery that knocking down PPARα can reduce high glucose-induced cardiomyocyte pyroptosis and revealed the regulatory relationship between PPARα and GDF11 in DCM.

**Fig. 7:**
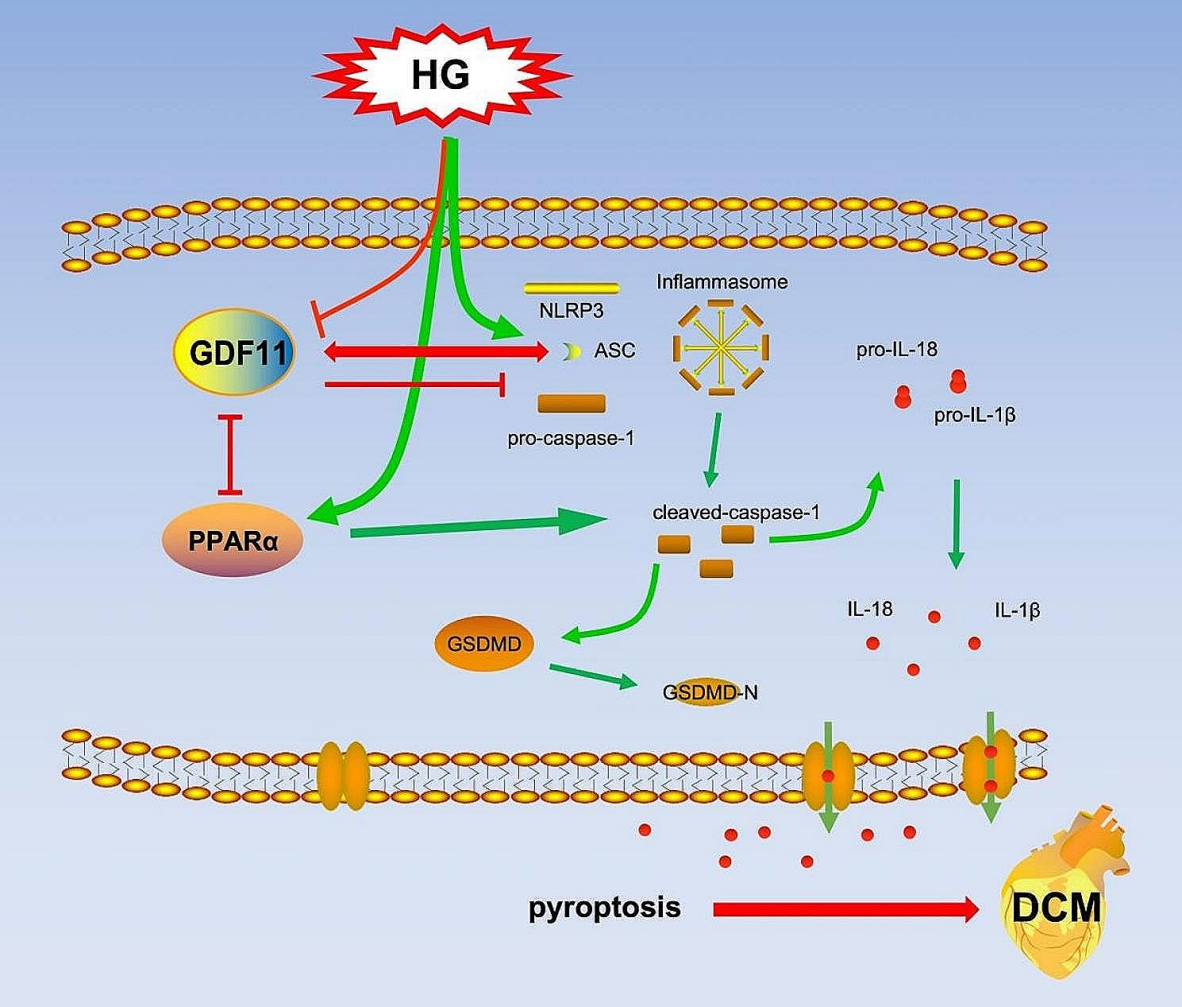
Treatment with HG can activate inflammasomes in cardiomyocytes and activate PPARα, while decreasing the expression of GDF11. Under HG treatment, GDF11 can inhibit inflammasome activation and thereby suppress pyroptosis by binding to ASC. The expression of GDF11 is regulated by PPARα under HG treatment, and PPARα can promote cardiomyocytes pyroptosis

The conclusions of this study are as follows: (1) GDF11 can inhibit high glucose-induced pyroptosis in H9c2 cardiomyocytes and pyroptosis in the hearts of mice with diabetic cardiomyopathy (DCM). (2) GDF11 blocks inflammasome activation by binding with ASC. (3) The PPARα/GDF11/ASC pathway is involved in regulating cardiomyocyte pyroptosis in DCM (Fig. 7). GDF11 has been a subject of interest as an aging-related molecule, and its function in the heart has been controversial, which may be closely related to its altered expression with age [42]. Several researchers have different views on the role of GDF11 in regulating myocardial injury [17, 19, 43]. However, many studies mainly focus on the systemic GDF11 rather than the expression changes of myocardial-specific GDF11. It is also important to consider the age of individuals in disease states, as well as whether the disease is chronic or acute. In comparison to other myocardial diseases, the pathogenesis of DCM is mainly attributed to the heart being in a state of long-term high blood sugar and chronic inflammation, indicating adistinct pathogenesis. Therefore, to more precisely prevent DCM, research on myocardial-specific targets is essential. Our results indicate that the cardiac-specific overexpression of GDF11 can improve DCM symptoms by reducing myocardial cell death and fibrosis, which is consistent with its function in promoting angiogenesis in diabetes [44]. Our study findings suggest that cardiac-specific activation of GDF11 may offer a promising therapeutic approach for preventing cardiomyopathy in diabetic patients. Therefore, targeting the heart specifically to enhance GDF11 expression could potentially serve as a clinical treatment strategy for DCM in the future. And it is also critical to investigate the impact of GDF11 on clinical outcomes across diverse age groups. In summary, our current research provides the first evidence that GDF11 exerts antipyroptotic effects and ameliorates DCM through the PPARα/GDF11/ASC axis. This finding may offer new insights for the treatment and study of DCM in the future.

### Electronic supplementary material

Below is the link to the electronic supplementary material.

Additional file 1:

## Data Availability

The data used and/or analyzed during the study are available from the corresponding author on reasonable request.

## Abbreviations

GDF11: Growth differentiation factor 11
DCM: Diabetic cardiomyopathy
ASC: Apoptosis-associated speck-like protein containing a CARD
PPARα: Peroxisome proliferator-activated receptor α
GSDMD: Gasdermin D
PRRs: Pattern recognition receptors
STZ: Streptozotocin
HFD: High-fat diet
LV: Left ventricle
LVEF: LV ejection fraction
FS: Fractional shortening
LVIDd: LV internal diastolic diameter
IVSd: Interventricular septal width during enddiastole
LVPW: LV posterior wall
PYD: PYRIN domain
NLRP3: NOD-like receptor (NLR) family pyrin domain containing 3
