# Supplementary material for: Growth differentiation factor 11 regulates high glucose-induced cardiomyocyte pyroptosis and diabetic cardiomyopathy by inhibiting inflammasome activation
Source: Cardiovasc Diabetol. 2024 May 7;23:160. doi: 10.1186/s12933-024-02258-3 (PMC11077721; doi:10.1186/s12933-024-02258-3)
Supplement: Supplementary file 1 — Additional file 1: [file 12933_2024_2258_MOESM1_ESM.docx]

**Growth differentiation factor 11 regulates high glucose-induced cardiomyocyte pyroptosis and diabetic cardiomyopathy by inhibiting inflammasome activation**

Jing Zhang,^1-7^ Guolong Wang,^1-7^ Yuxuan Shi,^1-7^ Xin Liu,^1-7^ Shuang Liu,^1-7^ Wendi Chen,^1-7^ Yunna Ning,^1-7^ Yongzhi Cao,^1-7^ Yueran Zhao,^1-7,*^ Ming Li^1-7,*^

**Author affiliations:**

^1^State Key Laboratory of Reproductive Medicine and Offspring Health, Center for Reproductive Medicine, Institute of Women, Children and Reproductive Health, Shandong University, 250012, China

^2^National Research Center for Assisted Reproductive Technology and Reproductive Genetics, Shandong University, Jinan, Shandong, 250012, China

^3^Key Laboratory of Reproductive Endocrinology (Shandong University), Ministry of Education, Jinan, Shandong, 250012, China

^4^Shandong Technology Innovation Center for Reproductive Health, Jinan, Shandong, 250012, China

^5^Shandong Provincial Clinical Research Center for Reproductive Health, Jinan, Shandong, 250012, China

^6^Shandong Key Laboratory of Reproductive Medicine, Shandong Provincial Hospital Affiliated to Shandong First Medical University, Jinan, Shandong, 250012, China

^7^Research Unit of Gametogenesis and Health of ART-Offspring, Chinese Academy of Medical Sciences (No.2021RU001), Jinan, Shandong, 250012, China

^*^Address correspondence to: Yueran Zhao, PhD, Center for Reproductive Medicine, Shandong University, Jinan, Shandong, 250012, China. Email: [yrzhao@sdu.edu.cn](mailto:yrzhao@sdu.edu.cn) Ming Li, Dr, Center for Reproductive Medicine, Shandong University, Jinan, Shandong, 250012, China. Email: drmingli@yeah.net

**Supplementary Table 1: Antibodies used**

| Antibody | Dilution | Company |
| --- | --- | --- |
| GDF11 | 1:1000(WB); 1:100(IHC/IF) | Abcam (ab124721) |
| NLRP3 | 1:1000(WB); 1:100(IHC/IF) | Abcam (ab263899) |
| ASC | 1:1000(WB); 1:100(IHC/IF) | Proteintech (10500-1-AP) |
| c-caspase-1 | 1:1000(WB); 1:100(IHC/IF) | Affnity (#AF4005) |
| GSDMD-N | 1:1000(WB); 1:100(IHC/IF) | Affnity (#DF13758) |
| β-Actin | 1:1000(WB) | Abclonal (AC028) |
| α-Actinin | 1:100(IHC/IF) | Cell Signaling Technology  (#69758) |
| PPARα | 1:1000(WB); 1:100(IHC/IF) | Invitrogen (PA1-822A) |
| collagenase III | 1:100(IHC/IF) | Abcam (ab7778) |
| HA | 1:1000(WB) | Proteintech (66006-2-Ig) |
| c-Myc | 1:1000(WB) | Proteintech (10828-1-AP) |

**Supplementary Table2: Blood glucose in mice in the indicated timeline.**

| Blood glucose (mmol/L) | NC | DCM | DCM+  AAV9-NC | DCM+  AAV9-GDF11 | DCM+  AAV9-GDF11+  PPARα agonist | DCM+  AAC9-NC+  PPARα agonist |
| --- | --- | --- | --- | --- | --- | --- |
| baseline | 7.48+0.97 | 6+1.33* | 8.24+0.90* | 6.57+1.63* | 6.27+1.55* | 7.51+0.56* |
| 1w | 7.53+1.08 | 18.07+1.05* | 18.44+1.79* | 18.87+0.39* | 18.7+1.02* | 19.13+1.40* |
| 5w | 8.57+0.81 | 22.3+1.81* | 22.51+3.75* | 23.08+3.45* | 22.99+3.49* | 25.3+3.18* |
| 9w | 7.16+0.85 | 24.56+2.15* | 25.19+4.58* | 22.83+3.94* | 22+7.61* | 26.67+1.00* |
| 13w | 7.81+1.25 | 29.04+3.06* | 29.64+2.79* | 24.87+2.51* | 27.41+2.66* | 28.5+3.48* |
| 17w | 6.99+0.94 | 27.76+2.38* | 28.56+4.03* | 24+2.59* | 27.79+1.01* | 28.66+1.48* |

Data are presented as the mean ± SD. P values were calculated using a one-way analysis of variance test and LSD test was used for multiple comparisons. Data are expressed as the mean ± SD. *P < 0.05 vs. NC.

 **Figure S1:** **GDF11 inhibits pyroptosis, that is reliant on the activation of the inflammasome, in high-glucose-treated H9c2 cells.** (A) Forced expression of ASC using the eukaryon expression plasmid with EF1α promoter in cardiomyocytes (n=3 per group). Forced expression of ASC sensitized cardiomyocytes to undergo pyroptosis when exposed to high glucose for 36h compared with the control. Pyroptosis was detected by PI assay (B) (n=3 per group) and western blot analysis of pyroptosis-associated proteins (GSDMD-N, c-caspase-1, IL-1β, ASC, NLRP3) (C-D) (n=3 per group). Scale bar, 50μm; (E) GDF11 was knocked down by using the small interfering RNA (n=3 per group). Knockdown of GDF11 sensitized cardiomyocytes to undergo pyroptosis when exposed to high glucose for 36h compared with the control. Pyroptosis was detected by PI assay (F) (n=3 per group) and western blot analysis of pyroptosis-associated proteins (GSDMD-N, c-caspase-1, IL-1β, ASC, NLRP3) (G-H) (n=3 per group). Scale bar, 50μm. Data are presented as means ± standard deviation from at least three independent experiments. **P < 0.01, ***P < 0.001, ****P<0.0001. Statistical analysis was carried out by Student’s t-test.

**Figure S2: GDF11 ameliorates fibrosis, and pyroptosis in DCM mice.** (A) Collagen deposition detected by immunohistochemical of Col III (*n* = 5 per group). (B-E) Immunofluorescence staining was used to detect the expression of GDF11 (B) and pyroptosis-associated proteins (c-caspase-1, NLRP3, IL-1β) (C-E) in mice heart (n=5 per group). (F) Immunohistochemical assay of pyroptosis-associated proteins (ASC, c-caspase-1, IL-1β) in mice heart (n=5 per group). Data are presented as means ± standard deviation from at least three independent experiments. ****P < 0.0001. Statistical analysis was carried out by Student’s t-test.

**Figure S3: Overexpression of PPARα inhibits GDF11 and promotes high-glucose-induced pyroptosis of cardiomyocytes.** (A) Forced expression of PPARα using the eukaryon expression plasmid with EF1α promoter in cardiomyocytes (n=3 per group). Forced expression of PPARα sensitized cardiomyocytes to undergo pyroptosis when exposed to high glucose for 36h compared with the control. Pyroptosis was detected by PI assay (B) (n=3 per group) and western blot analysis of pyroptosis-associated proteins (GSDMD-N, c-caspase-1, IL-1β, ASC, NLRP3) (C-D) (n=3 per group). Data are presented as means ± standard deviation from at least three independent experiments. **P < 0.01, ***P < 0.001. Statistical analysis was carried out by Student’s t-test.

**Figure S4: PPARα reverses the ameliorative effects of GDF11 on fibrosis and pyroptosis in the hearts of DCM mice.** (A) Collagen deposition detected by immunohistochemical of Col III (n=5 per group). (B-E) Immunofluorescence staining was used to detect the expression of pyroptosis-associated proteins (c-caspase-1, IL-1β) (B) in mice heart (n=5). Data are presented as means ± standard deviation from at least three independent experiments. ****P<0.0001. Statistical analysis was carried out by Student’s t-test.
